# Chemical Characterization of Hot Trub and Residual Yeast: Exploring Beer By-Products for Future Sustainable Agricultural Applications

**DOI:** 10.3390/foods14122081

**Published:** 2025-06-13

**Authors:** Laura Alessandroni, Riccardo Marconi, Marco Zannotti, Stefano Ferraro, Tereza Dolezalova, Diletta Piatti, Ghazal Namazzadeh, Simone Angeloni, Gianni Sagratini

**Affiliations:** 1 School of Pharmacy, Chemistry Interdisciplinary Project (ChIP), University of Camerino, Via Madonna delle Carceri, 62032 Camerino, Italy; laura.alessandroni@unicam.it (L.A.); riccardo.marconi@studenti.unicam.it (R.M.); diletta.piatti@unicam.it (D.P.); ghazal.namazzadeh@unicam.it (G.N.); gianni.sagratini@unicam.it (G.S.); 2 School of Science and Technology, Chemistry Division, Chemistry Interdisciplinary Project (ChIP), University of Camerino, Via Madonna delle Carceri, 62032 Camerino, Italy; marco.zannotti@unicam.it (M.Z.); stefano.ferraro@unicam.it (S.F.); 3 Department of Food Analysis and Nutrition, University of Chemistry and Technology, Prague, Technicka 3, 166 28 Prague, Czech Republic; tereza1.dolezalova@vscht.cz

**Keywords:** hot trub, beer, brewer residual yeast, circular economy, beer by-products, agricultural applications

## Abstract

Three types of solid waste are produced during beer fermentation: spent grain, hot trub, and residual yeast. While the first is used as livestock feed, the seconds has not yet found any real reapplication in the field of circular economy. The aim of this work is to study and characterize these two brewing wastes, i.e., hot trub and residual yeast, to evaluate their potential reuse in the agricultural field. Samples from top-fermented and bottom-fermented beers were chemically investigated. Initially, the safety was assessed via multi-detection analysis of 57 mycotoxins, and all samples were deemed safe. Subsequently, the chemical and elemental composition was examined via ICP-MS and microanalysis, along with phenolic compounds and antioxidant activity via HPLC and spectrophotometric determinations, to achieve a thorough characterization of these waste samples. The C/N ratio of residual yeast from top-fermented beer and hot trub of the bottom-fermented one were near the optimal one (10:1). This research marks an initial step towards repurposing brewery waste materials as fertilizers. The subsequent steps will involve the formulation and field trials.

## 1. Introduction

Beer is an alcoholic beverage made from four fundamental ingredients: water, malt, hops, and yeast. The brewing process generates large quantities of three solid wastes: spent grain, hot trub, and residual yeast [1,2]. Brewers’ spent grain (BSG) is the residual malt grain that remains after the mashing process. BSG has already been studied for various applications, such as animal feed, biogas production and even food production [3,4]. However, other two types of solid waste are produced in the subsequent beer-producing steps, namely hot trub (HT) and brewer’s residual yeast (RY), which have not yet found significant fields of reapplication. The first, HT, consists of solid and particulate matter formed during wort boiling and subsequently separated through the whirlpool process. It contains a mixture of hop particles, entrained wort, and high molecular weight colloidal proteins that tend to coagulate during the wort boiling process [1]. The composition of HT is influenced by numerous factors including the varieties of barley and hops, concentration, solubilization degree of substances from hops and malt, and brewing process setups, such as pH control, ion concentration, homogenization time, and oxidation during cooking, which all play a central role in the residue composition. A large portion of hop compounds (approximately 85%) is insoluble in the wort and is discarded as HT [5]. HT is constituted by 50–60% protein, 15–20% resins, 2–3% ash, and 1–2% fatty acids [6]. Despite these beneficial nutrients, it is unsuitable for consumption by humans or animals due to its unpalatable bitter taste [7,8]

The second brewing process by-product is the RY, which forms when yeast reaches the end of its fermentative activity. The microbial biomass quantity is contingent upon several factors, including fermentation conditions, such as aeration, temperature, pH, inoculum concentration, as well as cell viability and vitality. The type of microorganism and the composition of brewer’s wort also have an impact on RY composition. The yeast used in brewing can be divided into two main categories: top-fermenting ‘ale’ yeasts (*Saccharomyces cerevisiae*) and bottom-fermenting ‘lager’ yeasts (*Saccharomyces pastorianus* and *Saccharomyces carlsbergensis*). The yeast strains selection significantly affects the organoleptic characteristics of the beer [9]. At the end of fermentation, the yeast is separated from the bulk liquid through the flocculation process. The yeast cells gather to form flocs, which rise to the top (in top-fermentation) or settle at the bottom of the vessel (in bottom-fermentation). Recent researches have begun to explore the potential reuses of brewing wastes in various sectors, including food and nutraceutical applications. For example, Sterczyńska et al. (2021) characterized hot trub for its rheological and microbiological properties, highlighting its potential for reuse in brewing or food processes [10]. Additionally, bioactive flavonoids, such as xanthohumol, found in hops and brewing residues, have attracted attention for their health-promoting properties and extraction potential [11]. These studies underscore a growing interest in valorizing brewing by-products, further supporting the relevance of exploring their use in sustainable agricultural applications.

In 2022, the European Union Member States produced a total of 34.3 billion liters of alcoholic beer and nearly 1.6 billion liters of low or non-alcoholic beer, averaging almost 80 L per capita (European Statistical Office, 2023) [12]. This level of production results in the generation of substantial quantities of by-products and wastes. In fact, around 14–19 kg of BSG, 0.2–0.4 kg of HT and 2–4 kg of RY are generated each hectoliter of beer [3,13,14]. It follows that in the EU in 2022, the brewery industry produced approximately 5–7 million tons of BSG, 72–140 mils tons of HT and 700–1400 mils tons of RY. Nowadays, according to sustainability and circular economy policies, the attention is focused on the possibilities of reusing waste and by-products. For this reason, in accordance with the waste hierarchy outlined in Article 4 of the EU revised waste framework (Directive 2008/98/EC; European Union, 2008) [15], the primary goal of this study was to support European principles by promoting waste management practices to prevent harm to the environment or human health. By identifying new applications that enhance their value and reduce waste, this research supports a more circular and resource-efficient food production model. Moreover, improving the sustainability of upstream processes, such as raw material cultivation, indirectly contributes to the overall sustainability and resilience of the food supply chain. Converting hot trub (HT) and residual yeast (RY) from conventional waste disposal to agricultural applications would offer notable environmental benefits. This approach can significantly reduce the volume of organic waste generated by breweries, lowering disposal costs and environmental impact. Moreover, repurposing these by-products contributes to nutrient recycling by returning valuable macro- and microelements to the soil, thereby supporting more sustainable and circular agro-industrial systems [12,13].

To the best of authorsknowledge, few scientific evidences are present about the reuse of these wastes, and none have reported their application to improve soil quality. This work aims to determine whether the direct application of these by-products to soil could function as a soil enricher or fertilizer for barley cultivation, thereby supporting the full circularity of beer production chain. Therefore, the safety of HT and RY from two differently fermented beers was assessed by monitoring 57 mycotoxins by UHPLC-MS/MS, and they were chemically characterized by ICP-MS, microanalysis, HPLC, and spectrophotometric determinations.

## 2. Materials and Methods

### 2.1. Samples

**Figure 1:**
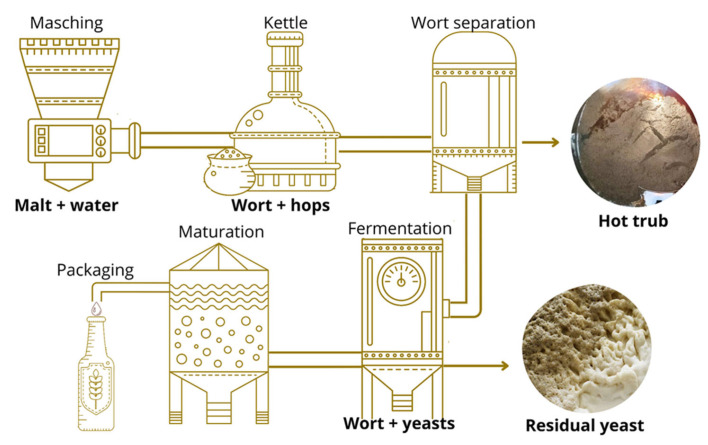
Graphic representation of beer brewing process and by-product collection steps.

The samples were provided by the Blink Brewery (Pesaro, PU, Italy), a specialized company in the production of craft beers. Two HT and two RY samples were collected from the production process of two types of beer, one bottom- and one top-fermented. A total of four groups of samples were analyzed: bottom-fermented hot trub (HTBF), bottom-fermented residual yeast (RYBF), top-fermented hot trub (HTTF), and top-fermented residual yeast (RYTF). The beers were selected by observing two criteria: firstly, the fermentation style and secondly the amount of hops used. The bottom-fermented beer was produced as a Helles-style beer and had a moderate alcohol content (4.8% ABV) with herbaceous and floral hop aromas. In contrast, the top-fermented beer was produced as a double IPA style beer and had a high alcohol content (8% ABV) and generous hopping that produced tropical hints. The specific characteristics of the samples are summarized in Table S1. The specific collection steps during the brewing process are outlined in Figure 1. The hot trub samples were collected during the whirlpool process (after the transfer of the wort into the fermenter) while the brewer residual yeast samples were taken at the end of fermentation. The samples were filtered under vacuum, and, for drying steps, two strategies were adopted. HT, as originating from a boiling process, was dried in an oven at 80 °C until constant weight while RY was placed in a desiccator at 45 °C until constant weight, to preserve its characteristics. Finally, each sample was stored at −20 °C until use.

### 2.2. Chemicals and Reagents

Folin–Ciocalteu phenol reagent (FC), sodium carbonate anhydrous (≥99%, Na_2_CO_3_), gallic acid (analytical standard, ≥97.5–102.5%, C_7_H_6_O_5_), sodium nitrite (≥99.0%, NaNO_2_), aluminum chloride (≥99%, AlCl_3_), sodium hydroxide (≥98%, NaOH), rutin (≥94%, C_27_H_30_O_16_), 6-hydroxy-2,5,7,8-tetramethylchromane-2-carboxylic acid (≥97.0%, trolox, C_14_H_18_O_4_), 2,2-diphenyl-1-picrylhydrazyl (DPPH, C_18_H_12_N_5_O_6_), and the analytical standards of the phenolic compounds, which include ursolic acid, oleanolic acids, procyanidin A2, procyanidin B2, rutin, quercetin-3-D-galactoside, gallic acid, (+)-catechin hydrate, (−)-epicatechin, phloretin, phlorizin, chlorogenic acid, neochlorogenic acid, caffeic acid, p-coumaric acid, trans-ferulic acid, cyanidin-3-glucoside, kaempferol, and quercetin were supplied by Merck (Milan, Italy), except for kaempferol-3-glucoside, which was purchased from PhytoLab (Vestenbergsgreuth, Germany). A total of 57 certified mycotoxin standards and their metabolites (Table S2) were obtained from Cayman Chemical (Ann Arbor, MI, USA), LKT Laboratories (St. Paul, MN, USA), Merck (Darmstadt, Germany), Romer labs (Getzersdorf, Austria) and Toronto Research Chemicals (Toronto, ON, Canada). Namely, the standards were 22 *Fusarium* toxins (nivalenol, deoxynivalenol, deoxynivalenol-3-glucoside, fusarenon X, neosolaniol, 3- and 15-acetyldeoxynivalenol, diacetoxyscirpenol, HT-2 and T-2 toxins, verrucarol, fumonisins B1, B2 and B3, zearalenone, α- and β-zearalenol, enniatins A, A1, B and B1 and beauvericin); 17 *Aspergillus* and *Penicillium* toxins (aflatoxins B1, B2, G1 and G2, ochratoxin A, citrinin, cyclopiazonic acid, sterigmatocystin, patulin, gliotoxin, meleagrin, mycophenolic acid, paxilline, penicillic acid, penitrem A, roquefortine C, verruculogen); 12 ergot alkaloids produced by *Claviceps* (agroclavine, ergosine, ergosinine, ergocornine, ergocorninine, ergocryptine, ergocryptinine, ergocristine, ergocristinine, ergotamine, ergotaminine, ergometrine); 1 *Stachybotrys* toxin (stachybotrylactam); 1 *Phomopsis* toxin (Phomopsin A) and 4 *Alternaria* mycotoxins (alternariol, alternariol-monomethylether, tentoxin and tenuazonic acid). A composite working mixture of all mycotoxins at a concentration of 1000 µg/L in acetonitrile was prepared for the purpose of calibration and spiking experiments. It has been stored at −20 °C and conditioned to room temperature before use. The purity of all standards was in the range of 95.0–99.0%. Hydrogen peroxide solution 30% and HPLC-grade methanol were supplied by Carlo Erba (Milan, Italy). HPLC-grade formic acid (99%) was purchased from Merck (Darmstadt, Germany). Ultrapure water was obtained from the Milli-Q SP Reagent Water System (Millipore, Bedford, MA, USA). Before HPLC analysis, all solutions and samples were filtered with Captiva PTFE 13 mm, 0.45 μm syringeless filters which were purchased from CPS analitica (Milan, Italy).

### 2.3. Quantification of Mycotoxins

The multi-detection analysis of 57 mycotoxins in our four different samples was performed using UHPLC-QTRAP-MS/MS, following a procedure and analytical method reported in a previous study [16]. The mycotoxins investigated were *Fusarium* toxins (nivalenol, deoxynivalenol, deoxynivalenol-3-glucoside, fusarenon X, neosolaniol, 3- acetyldeoxynivalenol and 15-acetyldeoxynivalenol, diacetoxyscirpenol, HT-2 and T-2 toxins, verrucarol, fumonisins B1, B2, and B3, zearalenone, α- and β-zearalenol, enniatins A, A1, B, and B1, and beauvericin), *Aspergillus* and *Penicillium* toxins (aflatoxins B1, B2, G1 and G2, ochratoxin A, citrinin, cyclopiazonic acid, sterigmatocystin, patulin, gliotoxin, meleagrin, mycophenolic acid, paxilline, penicillic acid, penitrem A, roquefortine C, verruculogen), mycotoxins produced by *Claviceps* (agroclavine, ergosine, ergosinine, ergocornine, ergocorninine, ergocryptine, ergocryptinine, ergocristine, ergocristinine, ergotamine, ergotaminine, ergometrine), *Alternaria* toxins (alternariol, alternariol-monomethylether, tentoxin and tenuazonic acid) and *Stachybotrys* and *Phomopsis* toxins (stachybotrylactam, phomopsin A). The standard references are reported in Table S2.

### 2.4. Chemical Composition Analysis

The chemical composition of hot trub and residual yeast was determined through the AOAC International procedures. The moisture content was determined by drying the sample in the oven (24 h, 133 °C) until a steady weight was achieved. The crude protein was obtained by the Kjeldahl method, and the total fat was estimated through the Soxhlet method; ash content was determined by incineration at 600 ± 15 °C. Total carbohydrate was estimated by difference.

### 2.5. Microanalysis and Elemental Composition by ICP-MS

To detect and quantify basic elements, including C, H, N and S, present in the samples, a Thermo Scientific™ FLASH 2000 CHNS/O Analyser (Waltham, MA, USA) was utilized. The dried samples were ground, weighed, and cautiously packaged in a tin capsule with vanadium pentoxide to be introduced into the instrument in solid form. Calibration samples were produced with the use of BBTO (2,5-Bis(5-tert-butyl-benzoxazol-2-yl)thiophene). The next step involved heating and combusting the samples in a furnace at a temperature of 950 °C with a constant flow of a helium stream of 140 mL/min in a temporarily enriched oxygen atmosphere (oxygen flow 250 mL/min). The combustion produced four reduced components: N_2_, CO_2_, H_2_O, and SO_2_. These components were separated in a chromatographic column and identified by the detector.

For elemental composition analysis, the dried hot trub and residual yeast powder (0.05 g) samples were mineralized by acid digestion using 4 mL 30% (*w*/*w*) H_2_O_2_, 1 mL 65% (*w*/*w*) HNO_3_, and 50 µL of a solution containing 2 mg/L of Au, Be, and Ru, added as a recovery standard. The digestion process was performed by a Berghof speedwave 4 microwave mineralizer using Teflon vessel. The mineralized samples were successively transferred into a plastic tube, diluted 1:10 with ultrapure water and then analyzed by ICP-MS Agilent Technologies 7500cx (Santa Clara, CA, USA). The ICP-MS analysis was performed using the operating conditions as follows: power 1550 W, carrier gas 0.9 L/min, make-up gas 0.00 L/min, sample depth 7 mm, nebulizer pump 0.1 r.p.s., and spray chamber temperature 2 °C. The ICP-MS instrumentation can work in the NoGas and in He mode; the latter condition, using a collision cell, permits overcoming polyatomic interference during the analysis. Internal standard solution was used for ICP-MS measurements; the isotope used as internal standard are as follows: ^45^Sc, ^115^In, ^140^Ce, and ^209^Bi (10 mg/L). The standard solutions of all the elements under investigations were prepared from standard stock solution (Agilent Technolgies, Santa Clara, CA, USA ISO 17034 [17]) by dilution with 1.0% HNO_3_ (Fluka Analytical, Aldrich, Milan, Italy). For the micro-elements (Li, Be, B, Al, Ti, V, Cr, Mn, Fe, Co, Cu, Zn, Ga, As, Se, Rb, Sr, Mo, Ru, Pd, Ag, Cd, Sn, Sb, Cs, Ba, Pb, U) the calibration curves were performed using the following standard solutions: 0.01 µg/L; 0.10 µg/L; 1.00 µg/L; 5.00 µg/L; 10.0 µg/L; 50.0 µg/L; 100.0 µg/L; and 500.0 µg/L. In the case of the macro-elements (Na, Mg, P, S, K, Ca), the following standard solutions were prepared: 0.50 mg/L; 1.00 mg/L; 2.50 mg/L; 5.00 mg/L; 10.0 mg/L; 25.0 mg/L; and 50.0 mg/L, for their calibration. In addition, the Hg element was calibrated using the following standard solutions: 0.1 µg/L; 0.5 µg/L; 1.0 µg/L; 5.0 µg/L; 10.0 µg/L.

### 2.6. Bioactive Phenols Monitoring

The extract preparation for each sample singularly was carried out by accurately grinding the dried matrix and adding a 70% ethanol solution. The matrix/solvent ratio was 1:10, and the extraction was performed in an ultrasonic bath (UAE) (FALC Instruments, Treviglio (BG), Italy) at a frequency of 40 kHz for 30 min. Each trial was performed in triplicate.

#### 2.6.1. Determination of the Total Phenolic Content

The content of total phenolic (TPC) of the extracts was determined by the Folin–Ciocalteu method reported by Piatti et al. (2024) with slight variations [18]. An extract aliquot of 0.5 mL or gallic acid solution (in calibration experiments) was mixed with 2.5 mL of 0.1 M Folin–Ciocalteu reagent. The solution was incubated in the dark for 5 min at room temperature. Then 7 mL of a 7.5% Na_2_CO_3_ solution was added, and the mixture was left for 2 h in the dark, then absorbance was measured at 765 nm with an Agilent Technologies spectrophotometer (Cary 8454 UV-Vis, Woburn, MA, USA). Results were calculated by comparing the absorbance of samples with the standard calibration curve of gallic acid. TPCs were expressed as mg of gallic acid equivalents (GAE)/g dry weight (dw). Analytical determinations of TPC were carried out in triplicate for each sample.

#### 2.6.2. Determination of the Total Flavonoid Content

Total Flavonoid Content (TFC) was determined following a method described by Alessandroni et al. (2024) [19]. Briefly, 0.5 mL of extract solution was mixed with 0.15 mL of a 0.5 M NaNO_2_ solution and 3.2 mL of methanol/water solution (30% *v*/*v*). After 5 min, 0.15 mL of a 0.3 M AlCl_3_⋅6H_2_O solution and, after another 5 min, 1 mL of a 1 M NaOH solution were added. The solution was mixed and incubated in the dark for 30 min at room temperature. The absorbance was measured at 506 nm using a spectrophotometer. The TFC standard calibration curve was prepared using a rutin standard solution with the same procedure as described above. The results of TFC were expressed as mg of rutin equivalents (RTE)/g dry weight (dw). Three independent experiments were performed, and all results presented are means ± standard deviation.

#### 2.6.3. Radical Scavenging Activity Assay

Radical 2,2-diphenyl-1-picrydrazyl (DPPH) was used to determine the free radical scavenging activity of the extract according to Giusti et al., (2017) with some modifications [20]. To prepare the solution, 0.5 mL of extract or standard solution (ascorbic acid) or blank (ethanol) was mixed with 4.5 mL of ethanolic solution of DPPH (0.1 mM). After 30 min of incubation in the dark at room temperature, the level of DPPH reduction was measured spectrophotometrically at 517 nm using the spectrophotometer. The reference antioxidant for the calibration curve of the DPPH method was trolox (6-hydroxy-2,5,7,8-tetramethylchroman-2-carboxylic acid). The percentage of inhibition of DPPH, calculated according to the Formula (1):

(1) % Inhibition = [(A0 − A1)/A0] × 100

where A0 is the absorbance value of the blank and A1 is the absorbance value of the sample.

#### 2.6.4. Polyphenols Quantification by HPLC-DAD

The extracts used for the quantification of polyphenols were prepared following the procedure reported in Section 2.6. Subsequently, each extract was totally dried under nitrogen gas flow. An aliquot of 50 mg of each dry extract was dissolved in 10 mL of ultrapure water. The analysis of 20 polyphenols was performed using a previously developed method with some modifications [21]. A 1260 Infinity HPLC system (Agilent Technologies, Santa Clara, CA, USA) coupled with a diode array detector (DAD) was used. The separation of the analytes was obtained using a Synergi Polar-RP C18 (4.6 mm × 250 mm, 4 µm) analytical column from Phenomenex (Cheshire, UK). For analyses, the mobile phase was a mixture of (A) water with 0.1% formic acid and (B) methanol with 0.1% formic acid, flowing at 1 mL/min in gradient conditions: 0 min, 20% B; 0–15 min, 20% B; 15–45 min, 100% B. The column temperature was set to 30 °C and the injection volume was 10 µL. UV spectra were recorded in the range of 210–400 nm for the 20 compounds, and the following wavelenghts were used for quantification: 210 nm for ursolic and oleanolic acids, 230 nm for procyanidin A2 and procyanidin B2, 265 nm for rutin, quercetin-3-D-galactoside and kaempferol-3-glucoside, 272 nm was used for gallic acid, 280 nm for (+)-catechin hydrate, (−)-epicatechin, phloretin, and phlorizin, 325 nm for chlorogenic acid, neochlorogenic acid, caffeic acid, p-coumaric acid, and trans-ferulic acid, 520 nm for cyanidin-3-glucoside, and 365 nm for kaempferol and quercetin. All analytes were quantified using the calibration curves obtained from the analytical standards injections.

### 2.7. Statistical Analysis

All the resulting data were collected in a database, which was uploaded in MetaboAnalyst 5.0 tool (https://www.metaboanalyst.ca/ accessed on 30 July 2024) for statistical analyses. Data were Log_10_ transformed and Pareto scaled before statistical analysis to normalize the values and account for the large variability among the selected parameters. To further analyses and better visualize the results, a heatmap representation with hierarchical clustering analysis was generated. Therefore, volcano plots were plotted for group comparisons and features with a minimum fold change (FC) of 1.5 and *p*-value < 0.05 were considered as differently abundant in the groups comparison.

## 3. Results and Discussion

### 3.1. Levels of Mycotoxins in Hot Trub and Residual Yeast

Numerous authors have studied the presence of mycotoxins in beer; however, few articles have evaluated their presence in by-products such as trub [22,23]. To use these by-products in agricultural field, it is important to assess the presence of dangerous substances such as mycotoxins. In our study, among 57 mycotoxins monitored in four different samples, no legislatively regulated mycotoxins were found, and only enniatins (ENN) were detected. ENN B and ENN B1 were detected in HTBF (92 and 39 µg/kg, respectively). In HTTF, only ENN B1 (9 µg/kg) was detected. None of the monitored mycotoxins were detected in the samples of RYBF and RYTF. The reason for the presence of ENN in hot trub samples could be related to their low polarity and high molecular weight. In fact, this class of mycotoxins usually concentrates in spent grains, trub, and fermentation sediment, with a very low chance of transferring into the beer [23]. These results are consistent with previously published papers [23,24,25,26]. In particular, Lago et al. (2022) reported the presence of these mycotoxins (ENN A, ENN A1, ENN B, ENN B1) in trub and in residual yeast from the ale brewing process [26]. It is worth noting that there are currently no regulations for ENN in food in the EU, and that the European Food Safety Authority (EFSA) has only established no-observed-adverse-effect levels (NOAELs) and a health-based guidance value, threshold of toxicological concern (TTC). The TTC value for the sum of ENN was determined to be 1.5 µg/kg of body weight per day and the NOAELs for ENN B and ENN B1 were established as 763 and 244 μg/kg of body weight per day for broiler chickens, and 674 and 216 μg/kg of body weight per day for laying hens, respectively [27]. However, these samples are not intended as food or feed but are considered for application to the soil. There are very few studies on the transfer of mycotoxins from soil to plants. One such study by Mertz et al. (1981) investigated the transfer of aflatoxin B1 from contaminated soil to lettuce seedlings [28]. The transfer was less than 1%, probably because the toxin had been strongly adsorbed to the loam. Mantle and Chow (2000) studied the transfer of ochratoxin A (OTA) applied to the soil where coffee plants were grown [29]. The transfer of OTA from the soil to the plants was also less than 1%. To our knowledge, no publications to date have directly addressed the soil-to-plant transfer of ENNs, although high ENN transfer is not expected. In conclusion, the samples of hot trub and residual yeast can be further explored for reuse in the context of the circular economy and food waste management.

### 3.2. Chemical Composition

The nutritional composition (moisture, total carbohydrates, fat, protein, and ash content) of the dried samples of hot trub and residual yeast is reported in Table 1.

The hot trub had an average protein content of 20 g/100 g, in particular, the data obtained for HTBF had a content of protein of 30.2 ± 1.3 g/100 g, which was higher than the HTTF (17.95 ± 0.89 g/100 g). This difference may be due to the processing or to the raw materials. Similar results were reported in previous studies [7,30].

Hot trub is predominantly composed of macromolecules belonging to the carbohydrate family. These derived from the breakdown of starch released from the grains during the malt boiling process, resulting in the formation of various types of sugars [7]. The data obtained for carbohydrates, in this study reveal the highest content of 72.39 ± 1.08 g/100 g in the top-fermented beer despite the bottom-fermented beer having a value of 59.67 ± 1.42 g/100 g. These results were similar to the data reported by Saraiva et al. (2022) [31], who used trub as a new ingredient to enrich pasta, but they show a greater difference compared to the data reported by Santos and Martins (2024) for trub from Pilsner beer [30]. The total fat content of hot trub ranged between 4.45 ± 0.53 and 4.93 ± 0.58 g/100 g for HTBF and HTTF samples, respectively [30]. According to Rachwał et al. (2020) this by-product from the top and bottom-fermented beers contained small amounts of ash, 2.90 ± 0.2 g/100 g and 2.92 ± 0.2 g/100 g, respectively [7].

The hot trub composition of Lager and Pilsner beers were found to be similar according to studies conducted by Sterczyńska et al. (2021) and Saraiva et al. (2019) [8,32]. The results obtained for residual yeast indicate a remarkable high protein content in RYTF (24.3 ± 1.1 g/100 g) and RYBF (52.4 ± 2.1 g/100 g) beer samples. The high concentration of protein is primarily due to the separation of the yeast interior from the corresponding cell wall components. Consequently, the solution retains a considerable amount of free amino acids, including glutamic acid, which significantly impacts the flavor [33]. Meanwhile, the total carbohydrates content was higher than the protein content for the RYTF beer, with a value of 49.90 ± 1.70 g/100 g. On the other hand, in the bottom-fermented beer, the total carbohydrates content was lower (31.96 ± 2.19 g/100 g) than the protein content. This difference is mainly influenced by the yeast inoculation rate; in this case, 500 billion cells were inoculated for the bottom-fermented beer, respect to the top-fermented one which has a value less than 50% (Table S1). Consequently, the residual yeast by-products had a low total fat content, with values for bottom-fermented and top-fermented of 3.33 ± 0.39 and 12.6 ± 1.2 g/100 g, respectively. The RYBF (5.89 ± 0.40 g/100 g) and RYTF (6.35 ± 0.43 g/100 g) contained relatively low levels of ash. Similar results were reported by Rachwał et al. (2020) and Mathias et al. (2015) in commercial beer from Brazil [7,34]. The moisture content goes from 6.42 to 6.85 g/100 g in residual yeast and from 1.83 to 2.67 g/100 g in hot trub. The high concentration of carbohydrates, particularly sugars, accounts for the moisture levels observed in the samples. This substantial sugar content complicates the process of extracting water using hot-air drying. Sugars form a caramelized layer on the material’s surface, which hardens and significantly slows the water diffusion from the interior to the exterior. This phenomenon has been noted by Michailidis and Krokida (2014) [35].

### 3.3. Microanalysis and Elemental Composition Results

Evaluating the levels of various elements is crucial for assessing the potential use of the material as a fertilizer or soil amendment. Elements such as carbon, hydrogen, sulfur and nitrogen are essential for plant growth and estimating their concentrations provides insights into their potential metabolic impact in different plant species. Carbon was the most abundant element in all analyzed samples followed by hydrogen with a percentage from 43.18 to 46.89% and from 6.32 to 6.74%, respectively (Table 1). An exception was represented by the sample of RYBT which has a higher level of nitrogen (8.15 ± 0.14%) than hydrogen. On the other hand, despite sulfur and nitrogen being present in smaller amounts they still play important roles in crop plants. Adequate nitrogen (N) levels can significantly improve plant performance and yield. Conversely, a lack of nitrogen can severely stunt growth and development. This essential element plays a key role in many processes critical to plant health and productivity, including growth, leaf area increase, biomass production, and root growth. By improving dry mass and increasing nutrient uptake and balance, nitrogen can greatly benefit overall plant health [36]. Sulfur (S) content in fertilizers is also important because it affects both crop yield and quality [37]. Therefore, in the tested samples nitrogen and sulfur range, respectively, from 2.97 to 8.15% and from 0.27 to 0.65%. The growth and development of plants are primarily facilitated by the elements of carbon (C) and nitrogen (N). The ratio of C/N serves as a gauge for nitrogen use efficiency (NUE) and is used as an input parameter in certain ecological and ecosystem models [38]. The results of this study, shown in Table 1, provide a good C/N ratio near the optimal range (10:1) [38] for HTBF (8.63 ± 0.89) and RYTF (12.74 ± 0.17). Moreover, the value obtained for HTTF was higher than the optimal range with a C/N ratio of 15.66 ± 1.08. Mathias et al. (2015) reported a RYBF similar C/N ratio for residual yeast from traditional Brazilian Pilsner beer, but they observed a lower C/N ratio for hot trub compared to our data for HTBF [34]. There are few reports in the literature about the determination of the C/N ratio in this matrix and the differences between the data may be explained by all variations present in the brewing method.

**Figure 2:**
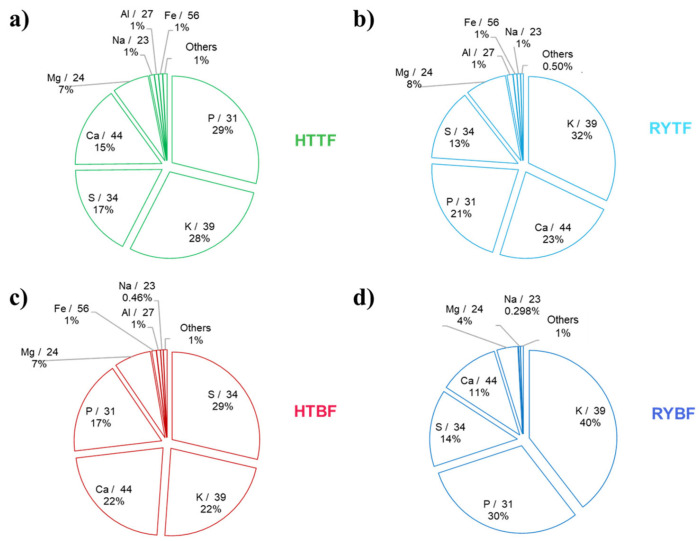
Element pattern of the freeze-dried samples by using ICP-MS. (**a**) Hot trub of top-fermented beer; (**b**) Residual yeast top-fermented beer; (**c**) Hot trub bottom-fermented beer; (**d**) Residual yeast bottom-fermented beer. The percentage of “others” represent the sum of percentages of elements that were found in concentration lower than 100 mg/Kg.

The results of elemental analysis conducted by ICP-MS technique are graphically resumed in Figure 2 and numerically explained in Table S3.

The elemental composition of the hot trub and residual yeast is dominated by five major elements, Mg, P, S, K, and Ca, which together account for 97% of the total sample mass. Notably, P and K, two of the most common and concentrated macroelements in fertilizers, range from 30% in HTBF, to 69% in RYBF. Analysis of the major elements shows that, in both hot trub and residual yeast, comparing the two-fermentation process, their concentrations are higher in samples from bottom-fermented beer, compared to those from top-fermented beer. In addition, for both the fermentation processes, the concentration of the macroelements P and K are enriched in the residual yeast, and this behavior is underlined in RYBF.

These results confirm a fertilizer-suitable minerals composition of all the analyzed samples. For Ca, Mg, and S, there are not any substantially differences between hot trub and residual yeast. Analyzing the results about the minority elements, in both the bottom and the top-fermented beer residuals, the presence of Zn, Fe, Mn, and Cu in high concentrations are always present. These elements are considered as microelements for plants for their proper growth and are essential for the morphology and anatomy [39], thus very important in the composition of a fertilizers. In this case, the concentration of those elements seems to be lower in the RYBF; an opposite trend for Fe was reported for the top-fermented beer, where the iron concentration is similar with between HTTF and RYTF.

### 3.4. Bioactive Phenolic Compounds Analysis

Polyphenolic compounds have been increasingly recognized for their potential biostimulant roles in agriculture. These molecules can influence plant growth by modulating hormone signaling pathways, enhancing stress tolerance, and stimulating microbial activity in the rhizosphere [40]. Moreover, their antioxidant properties may contribute to improved soil health by mitigating oxidative stress in both plants and beneficial soil microorganisms [41]. The results of total polyphenols, total flavonoids and antioxidant activity assays of the hot trub and residual yeast are shown in Table 2. The latter, reported as DPPH inhibition percentage, showed values from 27.38 ± 2.39 to 54.71 ± 1.11%, both referred to top-fermented beer by-products, HT and RY, respectively.

Our results show a higher inhibition percentage compared to the data reported by Fărcaş et al. (2013) for by-products obtained from a small-scale brewing process [42]. However, the data obtained for HTBF were similar to the data reported by Saraiva et al. (2019) before the extraction process of bitter compounds [8]. The TPC results varied from 0.40 to 1.65 mg GAE/g of dw, being HTBF and RYTF, respectively.

For RYBF, our data were comparable to that reported by León-González et al. (2018) who investigated the solid residual yeast of bottom-fermented beer provided by one of the largest beer producers in Spain [43]. Similar results emerged from previously published research [8,44]. Otherwise, a low level of flavonoids was found in all beer by-product samples. The range goes from 1.63 to 8.15 mg RTE/g of dw, in HTBF and RYTF, respectively. Comparing our results with previously published data on hot trub and residual yeast, they resulted similar to those reported by Senna Ferreira et al. (2021) [44]. Furthermore, León-González et al. (2018) reported similar data for residual yeast samples [43]. Specifically, RYBF showed similar results to solid residual yeast, and RYTF had similar results to liquid residual yeast from bottom-fermented beer provided by one of the largest beer producers in Spain. Among the analyzed samples the higher values in spectrophotometric assays were found in the RYTF sample. This could depend on the highest amount of hops (13.2 g/L) added during fermentation, in fact, the concentration of phenolic compounds in beer is heavily influenced by a range of factors, including the raw materials used in production, the brewing techniques, and the specific type of craft beer preparation protocols [45]. For example, the duration of hop boiling can vary depending on the beer style, leading to differences in phenolic compounds extraction from the hops and so their content in beer and by-products. These variables indicate that there are multiple contributing factors to the variation in phenolic content across different beer styles [42]. As such, the phenolic content of beer styles can be attributed to a combination of factors, including the strain of hops used, the brewing method, and hop boil time, which accounts for the differences observed in both samples and previous studies.

Spectrophotometric methods are able to give some general information regarding the estimation of global levels of polyphenolic substances. Therefore, profiling and quantification studies are largely practiced as preliminary measures. In the present study, a previously optimized HPLC-DAD method was involved to determine individual phenolic compounds in the hot trub and residual yeast of two different beer extracts, following the procedure outlined in Section 2.6.4. A total of twenty phenolic compounds were monitored, and among them, only gallic acid, kaempferol and trans-ferulic acid were quantified in all analyzed samples. This phenomenon could be related to the capacity of high molecular weight phenolic compounds to coprecipitate with the proteins and so be partially lost in the hot trub and spent yeast [46]. The sample with the highest content of polyphenols, among the monitored ones, was the HTTF with eleven phenolic compounds quantified through HPLC-DAD. The most abundant was phlorizin (13.80 mg/Kg), followed by oleanolic acid (12.15 mg/Kg), quercetin-3-D-galactoside (9.83 mg/Kg), and gallic acid (9.12 mg/Kg).

The major sources of polyphenols in beer and brewing by-products are malt and hops. Malt was reported to contain a variety of compounds, including proanthocyanidins, chlorogenic, caffeic, gallic, and p-coumaric acids. Hops flowers, on the other hand, contain approximately 4−14% of dry weight in polyphenols, which are mainly composed of phenolic acids, chalcones, flavonoids, and proanthocyanidins [47]. The results obtained for residual yeast samples are similar to the data obtained by León-González et al. (2018) who investigated the concentration of gallic acid, trans-ferulic acid, quercetin and kaempferol in the residual yeast of craft and industrial beers, reporting also a small amount of rutin and p-coumaric acid [43]. In this study, the investigation of the presence of polyphenols was necessary to understand if the hot trub and residual yeasts can be used in agriculture as fertilizers or soil improvers. Presented outcomes are partially in line with spectrophotometric assays results, this let hypothesize the presence of other phenolic derivatives which were not monitored through the applied method. Nowadays some research on the effect of phenolic compounds present in plants or plant extract shows some direct effects such as bioprotectant potential, biostimulant activity, defense mechanisms by modulation of enzymes, and regulation of phytohormone activity. While it is unlikely that phenolic compounds and extracts have a direct impact on proliferation or act as direct bioprotectants against pests and pathogens. Instead, their effects may be indirect, such as on the microbiomes of plants and soil or by eliciting induced disease resistance, which may assist in the bioprotectant or biostimulant functions. There is currently limited research on the collateral effects of externally applied phenolic chemicals and plant extracts [48]. On the other hand, numerous flavonoid compounds have been found to possess biological activities. These activities include promoting or inhibiting plant growth and nutrient absorption in a concentration-dependent manner, serving as signals in plant-microbial communication, as well as interacting with plant hormones. Plant phenols, including polyphenols and flavonoids, may control the C cycle through their effect on decomposer communities and eventual changes in their decomposing rates, hence on the C cycle. Soil organic matter represents the largest storage of soil C, N, P, S, Ca, Mg, and microelements. As a result, any changes in the mineralization or immobilization of C can have an impact on the availability of other essential nutrients [49].

### 3.5. Statistical Elaboration Results

**Figure 3:**
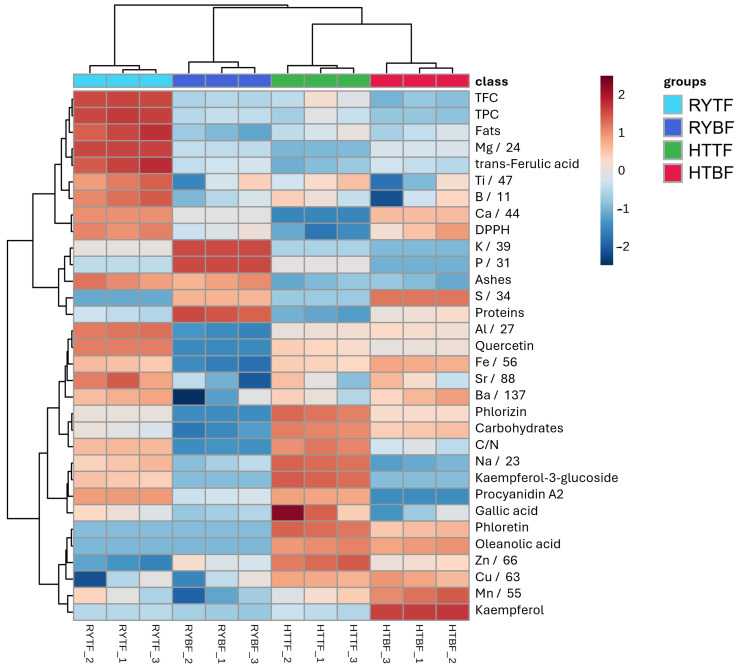
Heatmap representation and hierarchical clustering of the data resulting from the four groups analysis on hot trub and cold residual yeast from top- and bottom-fermented beers.

**Figure 4:**
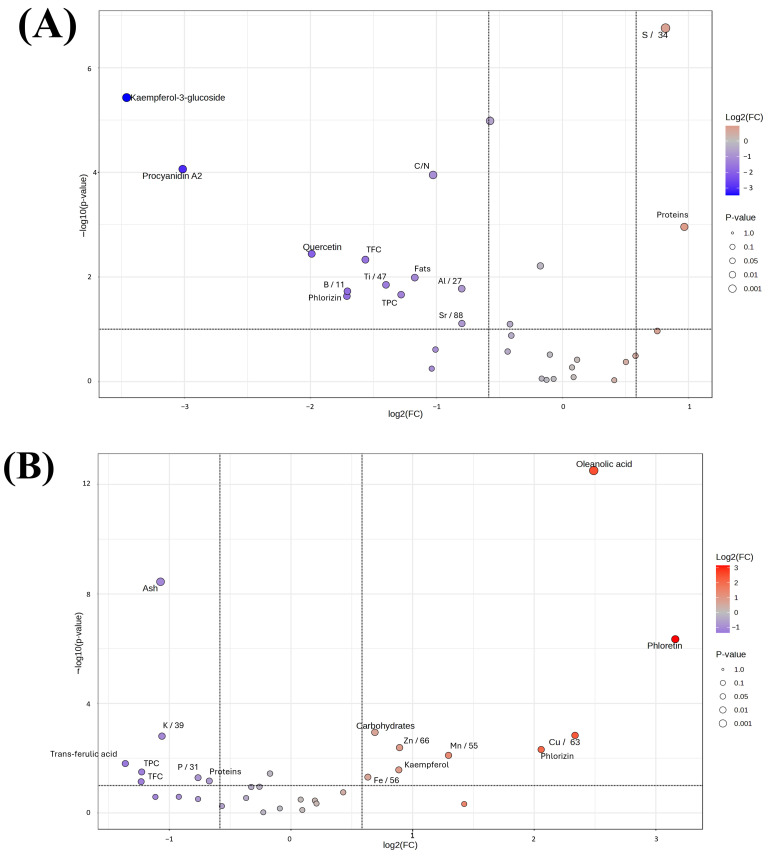
Volcano plot representing the expression of various characteristics and parameters for the four different samples. Comparison between top- and bottom-fermentation (**A**), and comparison between hot trub and residual yeast (**B**).

In order to distinguish the more appropriate beer waste to use as soil fertilizer for future applications, the relationship between the most important factors was investigated applying statistical elaboration of the data. The heatmap (Figure 3) provides a comprehensive hierarchical clustering analysis of different biochemical parameters among different samples, which reveals significant patterns and groupings. The samples are grouped into four clusters: green, orange, pink, and purple, while each cluster has a unique biochemical profile. In particular, the HTTF sample has higher values for parameters such as TPC, fats, and Cu, but low for Na and gallic acid. On the other hand, the RYTF samples have high values for parameters like ash, Fe, and DPPH, whereas TPC, phloretin, and phlorizin have low values. These variations are well visualized by the gradient of color from blue (low values) to red (high values). Moreover, some parameters, such as proteins, kaempferol, quercetin, and phloretin, are in close clusters, which means there are no significant differences among the samples, whereas some others, such as gallic acid and Na, have wider clusters. The sample of HTBF have the highest concentration of elements such as magnesium, iron, barium, sulfur, zinc, copper, and calcium while the RYBF sample showing a higher concentration only of potassium and phosphorus. The most abundant concentration of elements are presents in the RYTF samples due to eight different elements (Ba, Fe, Al, Sr, Ca, Mg, Ti, B, Na). To improve understanding of differences and amplify statistical significance, two distinct volcano plots were generated, each focusing on different sample attributes (Figure 4). The first graph examines the variations in fermentation type (TF vs. BF), while the second graph explores the divergence between the two types of beer waste (HT vs. RY).

The volcano plot comparing waste from top- and bottom-fermentation processes, reported in Figure 4A, reveals significant differences in the compounds’ abundance between the two. Procyanidin A2 is extremely abundant in the top-fermentation samples, with a high statistical significance, which is indicated by a *p*-value < 0.0000001. Kaempferol-3-glucoside also has a much lower concentration in bottom-fermented samples. While quercetin and phlorizin are more abundant in top-fermented samples with statistical significance. The C/N ratio presents with a moderate significance level and a slight decrease in the bottom-fermentation samples. Furthermore, compound classes, such as flavonoid (from TFC) and phenolic (from TPC), exhibit a moderate level of statistical significance and slight decreases in the bottom-fermentation samples. In summary, a differential profile of some compounds is found between wastes in both processes, and several compounds seem to be considerably higher in wastes from top-fermentation. The volcano plot of hot trub compared to residual yeast from the same brewing process shows very important differences in the content of certain compounds and elements (Figure 4B).

The most relevant difference is a statistically higher ash content in the residual yeast compared to hot trub. On the other hand, the hot trub shows an important amount of different compounds such as oleanolic acid, phloretin, phlorizin and other components, e.g., Cu and Mn. Some other compounds, such as trans-ferulic acid, TPC, and TFC, have no significant differences between hot trub and residual yeast. Potassium is slightly more abundant in the residula yeast, but the difference is not very important. In summary, there is a clear compositional difference between hot trub and residual yeast. Residual yeast contains significantly higher amounts of ash, whereas hot trub is enriched in oleanolic acid, copper, phloretin, phlorizin, and manganese. These differences highlight the unique chemical profiles of the two brewing residuals, suggesting distinct roles and potential applications within the brewing industry and beyond. In addition to their chemical composition, investigated in this work, hot trub and brewer’s residual yeast are highly biodegradable due to their rich organic composition, including proteins, lipids, and polysaccharides, which are readily utilized by soil microbial communities [50].

Finally, while the present study offers valuable insights into the chemical composition of hot trub and residual yeast from two differently fermented beers, the limited sample size represents an inherent constraint. These results should be viewed as preliminary, serving as a foundation for further investigations involving a more diverse range of samples and production contexts. Future studies should aim to include multiple batches and brewery sources to confirm the consistency and broader applicability of the observed trends.

## 4. Conclusions

This study is a pioneer in the analysis of waste products from beer production and reports the first and very encouraging results for the agricultural reuse of hot trub and residual yeast. Bringing the key findings of this research into the agricultural practice, hot trub and residual yeast exhibit encouraging possibilities for incorporation into farming systems, especially as organic soil enhancers, due to their chemical composition and C/N ratio. Their comparatively elevated levels of nitrogen, potassium and phosphorus indicate they may function as gradual nutrient providers. Moreover, due to their organic nature, these by-products are expected to be readily decomposed by soil microorganisms, likely boosting microbial activity and aiding soil fertility. However, their application rates should be optimized to avoid potential phytotoxicity or nutrient imbalances. Further studies focusing on field trials and long-term soil interaction using the product as such or in a formulation, would be valuable to fully assess their stability, mineralization rates, and effects on soil microbiota dynamics.

The implications of these findings are significant, as they offer valuable insights for stakeholders looking to foster a more sustainable brewing industry that prioritizes both ecological integrity and economic resilience.

## Figures and Tables

**Table 1:** Chemical composition and CHNS/O microanalysis results of the four groups of analyzed samples. Values expressed are means ± standard deviation of three parallel measurements. Elemental microanalysis data are expressed in percentages. Statistically significant differences between samples are expressed by different letters.

Compounds	Top-Fermented Beer		Bottom-Fermented Beer	
	Hot Trub (HTTF)	Residual Yeast (RYTF)	Hot Trub (HTBF)	Residual Yeast (RYBF)
Moisture (g/100 g)	1.83 ± 0.07 c	6.85 ± 0.25 a	2.67 ± 0.10 b	6.42 ± 0.23 a
Carbohydrates (g/100 g)	72.39 ± 1.08 a	49.90 ± 1.70 c	59.67 ± 1.42 b	31.96 ± 2.19 d
Proteins (g/100 g)	17.95 ± 0.89 d	24.3 ± 1.12 c	30.2 ± 1.34 b	52.4 ± 2.12 a
Total fats (g/100 g)	4.93 ± 0.58 b	12.6 ± 1.2 a	4.45 ± 0.53 b	3.33 ± 0.39 b
Ash (g/100 g)	2.90 ± 0.20 b	6.35 ± 0.43 a	2.92 ± 0.2 a	5.89 ± 0.40 a
Carbon (C) (% *w*/*w*)	46.44 ± 0.27	46.27 ± 0.57	46.89 ± 0.11	43.18 ± 0.19
Nitrogen (N) (% *w*/*w*)	2.97 ± 0.19 d	3.63 ± 0.003 c	5.46 ± 0.55 b	8.15 ± 0.14 a
Hydrogen (H) (% *w*/*w*)	6.74 ± 0.32	6.32 ± 0.13	6.66 ± 0.07	6.65 ± 0.01
Sulfur (S) (% *w*/*w*)	0.61 ± 0.35 a	0.27 ± 0.005 b	0.65 ± 0.06 a	0.52 ± 0.11 a
C/N Ratio	15.66 ± 1.08 a	12.74 ± 0.17 b	8.63 ± 0.89 c	5.30 ± 0.11 d

**Table 2:** Comparison of the total polyphenol content, total flavonoid content, antioxidant capacity, and polyphenolic composition of the four groups of beer by-products samples. Values expressed are means ± standard deviation of three parallel measurements. Single polyphenols results are expressed in mg/kg. Different superscript letters indicate a statistically significant difference (*p* < 0.05) between samples. (GAE: gallic acid equivalent, RT: Rutin equivalent; n.d. not detectable, dw: dry weight).

Compounds	Top-Fermented Beer		Bottom-Fermented Beer	
	Hot Trub (HTTF)	Residual Yeast (RYTF)	Hot Trub (HTBF)	Residual Yeast (RYBF)
DPPH (% Inhibition)	27.38 ± 2.39 ^c^	54.71 ± 1.11 ^a^	47.05 ± 6.06 ^ab^	39.25 ± 2.32 ^bc^
TPC (mg GAE/g of dw)	0.52 ± 0.12 ^b^	1.65 ± 0.06 ^a^	0.40 ± 0.01 ^b^	0.50 ± 0.02 ^b^
TFC (mg RTE/g of dw)	2.71 ± 0.60 ^b^	8.15 ± 0.09 ^a^	1.63 ± 0.16 ^c^	2.05 ± 0.03 ^bc^
Catechin	n.d.	2.17 ± 0.19	n.d.	n.d.
Gallic acid	9.12 ± 0.70 ^a^	8.17 ± 0.15 ^ab^	7.63 ± 0.40 ^b^	7.68 ± 0.08 ^b^
Kaempferol	1.32 ± 0.06 ^b^	1.28 ± 0.01 ^bc^	3.21 ± 0.05 ^a^	1.17 ± 0.03 ^c^
Kaempferol-3-glucoside	2.90 ± 0.27 ^a^	0.97 ± 0.09 ^b^	n.d.	n.d.
Oleanolic acid	12.15 ± 1.02	n.d.	10.96 ± 0.67	n.d.
Phloretin	4.60 ± 0.44 ^a^	n.d.	2.05 ± 0.19 ^b^	n.d.
Phlorizin	13.80 ± 1.51 ^a^	3.70 ± 0.07 ^b^	4.62 ± 0.07 ^a^	n.d.
Procyanidin A2	0.99 ± 0.05 ^b^	1.11 ± 0.1 ^a^	n.d.	0.22 ± 0.02 ^c^
Quercetin	1.37 ± 0.15 ^b^	3.31 ± 0.02 ^a^	0.99 ± 0.05 ^c^	n.d.
Quercetin-3-D-galactoside	9.83 ± 0.96	n.d.	n.d.	n.d.
*trans*-Ferulic acid	0.22 ± 0.02 ^b^	1.02 ± 0.11 ^a^	0.30 ± 0.02 ^b^	0.32 ± 0.015 ^b^

## Data Availability

The original contributions presented in the study are included in the article/Supplementary Material, further inquiries can be directed to the corresponding author.

## Supplementary Materials

The following supporting information can be downloaded at: https://www.mdpi.com/article/10.3390/foods14122081/s1, Table S1: Specific characteristics of hot trub and residual yeast samples; Table S2: List of certified mycotoxin standards; Table S3: Elemental composition by ICP-MS.
