# Supplementary material for: Chemical Characterization of Hot Trub and Residual Yeast: Exploring Beer By-Products for Future Sustainable Agricultural Applications
Source: Foods. 2025 Jun 13;14(12):2081. doi: 10.3390/foods14122081 (PMC12191850; doi:10.3390/foods14122081)
Supplement: Supplementary file 1 [file foods-14-02081-s001.zip › foods-3689323-supplementary.pdf]

Table S1. Specific characteristics of hot trub and residual yeast samples.

	Top-fermented beer		Bottom-fermented beer	
	Hot trub (HTTF)	*Residual yeast* (RYTF)	*Hot trub* (HTBF)	*Residual yeast* (RYBF)
Sampling temperature (°C)	90	2	90	2
Quantity of hops (g/L)	4.5	13.2	3.4	-
pH	5.21	4.34	5.19	4.55
International Bitterness Unit (IBU)	38	38	12	12
Rate of yeast inoculation (billions of cells)	-	250	-	550
Fermentation temperature/time (°C/ days)	-	18°C / 14 days	-	10°C / 14 days

- The sample is not subjected to treatment

Table S2. List of certified mycotoxin standards

No.	Mycotoxin	CAS	Producer
1	15-acetyldeoxynivalenol	88337-96-6	ROMER Labs Diagnostic GmbH
2	3-acetyldeoxynivalenol	50722-38-8	Merck Life Science spol. s r.o.
3	Aflatoxin B1	1162-65-8	Merck Life Science spol. s r.o.
4	Aflatoxin B2	7220-81-7	Merck Life Science spol. s r.o.
5	Aflatoxin G1	1165-39-5	Merck Life Science spol. s r.o.
6	Aflatoxin G2	7241-98-7	SIGMA-ALDRICH spol. s r.o
7	Agroclavine	548-42-5	TORONTO RESEARCH CHEMICALS,INC
8	Alpha-zearalenol	364-55-72-8	Cayman Chemical
9	Alternariol	641-38-3	Cayman Chemical
10	Alternariol-methylether	23452-05-3	Cayman Chemical
11	Beauvericin	26048-05-5	Cayman Chemical
12	Beta-zearalenol	71030-11-0	Cayman Chemical
13	Citrinin	518_75-2	Cayman Chemical
14	Cyclopiazonic acid	18172-33-3	Cayman Chemical
15	Deoxynivalenol	51481-10-8	Merck Life Science spol. s r.o.
16	Deoxynivalenol-3-glucoside	131180-21-7	ROMER Labs Diagnostic GmbH
17	Diacetoxyscirpenol	2270-40-8	ROMER Labs Diagnostic GmbH
18	Enniatin A	2503-13-1	Merck Life Science spol. s r.o.
19	Enniatin A1	4530-21-6	Cayman Chemical
20	Enniatin B	917-13-5	Merck Life Science spol. s r.o.
21	Enniatin B1	19914-20-6	Merck Life Science spol. s r.o.
22	Ergocornine	57432-60-7	ROMER Labs Diagnostic GmbH
23	Ergocorninine	564-37-4	ROMER Labs Diagnostic GmbH
24	Ergocristine	511-08-0	ROMER Labs Diagnostic GmbH
25	Ergocristinine	511-07-9	ROMER Labs Diagnostic GmbH
26	Ergocryptine	2706-66-3	ROMER Labs Diagnostic GmbH
27	Ergocryptinine	511-10-4	ROMER Labs Diagnostic GmbH
28	Ergometrine	60-79-7	ROMER Labs Diagnostic GmbH
29	Ergosine	561-94-4	ROMER Labs Diagnostic GmbH
30	Ergosinine	596-88-3	ROMER Labs Diagnostic GmbH
31	Ergotamine	113-15-5	ROMER Labs Diagnostic GmbH
32	Ergotaminine	639-81-6	ROMER Labs Diagnostic GmbH
33	Fumonisin B1	116355-83-0	Cayman Chemical
34	Fumonisin B2	116355-84-1	Cayman Chemical
35	Fumonisin B3	136379-59-4	LKT Laboratories
36	Fusarenon X	23255-69-8	ROMER Labs Diagnostic GmbH
37	Gliotoxin	67-99-2	Cayman Chemical
38	HT-2 toxin	26934-87-2	Merck Life Science spol. s r.o.
39	Meleagrin	71751-77-4	LKT Laboratories
40	Mycophenolic acid	24280-93-1	Cayman Chemical
41	Neosolaniol	36519-25-2	ROMER Labs Diagnostic GmbH
42	Nivalenol	23282-20-4	ROMER Labs Diagnostic GmbH
43	Ochratoxin A	303-47-9	Merck Life Science spol. s r.o.
44	Patulin	149-29-1	Apollo Scientific Ltd.
45	Paxilline	57186-25-1	Cayman Chemical
46	Penicillic acid	90-65-3	Cayman Chemical
47	Penitrem A	12627-35-9	Cayman Chemical

48	Phomopsin A	64925-80-0	Cayman Chemical
49	Roquefortine C	58735-64-1	LKT Laboratories
50	Stachybotrylactam	163391-76-2	TORONTO RESEARCH CHEMICALS,INC
51	Sterigmatocystin	10048-13-2	Cayman Chemical
52	T-2 toxin	21259-20-1	ROMER Labs Diagnostic GmbH
53	Tenuazonic acid	610-88-8	Cayman Chemical
54	Tentoxin	28540-82-1	LKT Laboratories
55	Verrucarol	2198-92-7	SIGMA-ALDRICH spol. s r.o
56	Verruculogen	12771-72-1	Cayman Chemical
57	Zearalenone	17924-92-4	Cayman Chemical

Table S3. Results of elemental composition analysis through ICP-MS, Values are expressed as average value (mg/kg) of the triplicate ± standard deviation.

Elements	Top-fermented beer		Bottom-fermented beer	
	Hot trub (HTTF)	*Residual yeast* (RYTF)	*Hot trub* (HTBF)	*Residual yeast* (RYBF)
Li / 7	0.11 ±3.85	0.14 ±1.12	0.09 ±2.31	0.08 ± 1.65
B / 11	6.24 ± 2.24	19.35 ± 3.36	4.32 ± 3.58	3.52 ± 1.25
Na / 23	171.75 ± 2.32	143.92 ± 4.23	100.61 ± 3.23	111.37 ± 5.53
Mg / 24	1371.02 ± 2.53	1766.39± 2.84	1480.16 ± 1.52	1446.90 ± 2.84
Al / 27	145.61 ±3.54	245.93 ± 5.24	152.86 ± 4.26	72.09 ± 3.45
P / 31	5789.46 ± 2.25	4894.72 ± 2.45	3783.47 ± 3.52	11362.08 ± 2.34
S / 34	3464.57 ± 5.61	3083.04 ± 4.23	6236.08 ± 5.63	5304.58 ± 4.55
K / 39	5739.03 ± 3.24	7407.63 ±3.02	4887.82 ± 2.56	14760.11 ± 2.98
Ca / 44	3044.62 ± 2.35	5220.50 ± 4.21	4795.78 ± 3.62	4161.73 ± 3.49
Ti / 47	5.63 ± 1.83	12.21 ± 2.42	2.14 ± 3.23	3.89 ± 2.56
Cr / 52	0.28 ± 3.23	0.41 ± 1.26	0.24 ± 2.26	0.12 ± 1.12
Mn / 55	20.04 ± 3.59	17.12 ± 5.3	41.47 ± 5.42	7.94 ± 3.24
Fe / 56	143.89 ± 5.14	157.97 ± 5.62	178.03 ± 4.52	49.74 ± 4.38
Co / 59	0.04 ± 4.60	0.12 ± 2.43	0.02 ± 4.89	0.07 ± 3.28
Ni / 60	0.23 ± 3.87	1.03 ± 2.08	0.29 ± 3.23	1.07 ± 1.60
Cu / 63	24.41 ± 2.29	4.61 ± 4.02	25.56 ± 5.52	5.29 ± 4.03
Zn / 66	88.02 ± 4.51	27.71 ± 2.32	54.27 ± 2.56	46.92 ± 4.04
As / 75	0.06 ± 1.61	0.11 ± 1.17	0.05 ± 1.93	0.12 ± 4.52
Se / 77	0.64 ± 4.09	0.80 ± 4.23	0.79 ± 5.23	0.62 ± 4.23
Sr / 88	13.54 ± 5.23	26.32 ± 4.26	15.38 ± 4.23	7.54 ± 3.22
Mo / 95	2.11 ± 3.37	0.75 ± 2.35	3.23 ± 3.30	2.48 ± 2.02
Pd / 105	0.10 ± 2.23	0.14 ± 1.81	0.10 ± 2.66	0.25 ± 1.52
Cd / 111	0.01 ± 2.35	0.01 ± 3.03	0.04 ± 5.23	0.03 ± 2.83
Sn / 118	0.03 ± 2.65	0.03 ± 3.12	0.03 ± 3.26	0.05 ± 3.41
Ba / 137	3.69 ± 1.95	8.84 ± 1.43	7.95 ± 2.6	0.71 ± 2.39
Hg / 202	1.35 ± 2.32	2.21 ± 3.25	1.86 ± 3.49	1.85 ± 3.91
Pb / 208	0.11 ± 2.25	0.15 ± 2.23	0.11 ± 3.52	0.09 ± 1.23
